# Hospital with Circular Wards

**Published:** 1884-06

**Authors:** 


### Selections.

---

HOSPITAL WITH CIRCULAR WARDS. Communicated by MR. J. BROWN, F.R.C.S.

For some time past, an attempt has been made to raise the funds needed to provide a hospital for the town and district of Burnley, Lancashire. The population exceeds 100,000 persons. In addition to the sum of £1,500, as the nucleus of an endowment fund, the committee have succeeded in obtaining subscriptions to the building fund to the extent of £12,600; and a site of two acres in extent has been given, along with a subscription of £1,000, by the executors of the late Colonel Hargreaves. Towards the building fund over £3,000 have been paid in by the working classes within a period of nine months. This has resulted from systematic collections made at the mills and workshops every four weeks. These collections the committee have suspended for the present, owing to the disturbed state of the cotton trade throughout the whole district, but they hope to be able to resume them ere long.

The plans approved of by the committee provide for the erection of a hospital with one-story pavilion wards of the circular form, as suggested by Professor Marshall, President of the Royal College of Surgeons. Two of these wards are to be built at present, with an internal diameter of sixty feet, for the accommodation of twenty beds each.

The architects, Messrs. W. Waddington & Son, of Burnley and Manchester, have designed a sun room, or day room, on the top of each ward, with a promenade twelve feet wide all round it. Access to the roof is given by a gently sloping spiral staircase surrounding the smoke and extraction flues, and occupying a closed-in space in the center of the ward, sixteen feet in diameter. There is then left for the ward itself an annular space twenty-two feet in width. The height of the ward at the eaves is fifteen feet, and at the inner portion sixteen feet. The roof is formed of concrete, supported on iron girders. The lineal wall space of the ward is 189 feet, giving an average wall space per bed, excluding doors, of about 8 feet 4 inches. The floor space is 132 square feet, and the cubic space 1,560 cubic feet per bed. Windows are placed between each bed, extending from two feet and a half from the floor to within a foot of the ceiling; and ample provision is made for ventilating, both at the floor level and at a height of about seven feet. From the inner part of the ceiling, and also from the floor, ventilating outlets are led to an extraction shaft which encircles the smoke flues.

The walls of the wards, water-closets, and bath-rooms are to be of glazed bricks or tiles. The floors of the wards are to be of oak, to be beeswaxed and polished. Separated from each ward by a cross-ventilated corridor are the bath-rooms and water-closets.

Connecting the circular wards with the main corridor is a well-ventilated passage, on either side of which is a single-bedded separation ward; and between these wards and the circular ward are the nurses' room and ward scullery.

A subway is provided from the cellar floor of the administrative block to the wards; in this all pipes will be laid; and along it coal, foul linen, cinders, and sweepings will be conveyed.

Provision is made on the site for the addition of four other wards similar in size to those described, and also for a children's ward for fourteen beds, intended to be forty feet in diameter, to have no central staircase, a sun-room, and to have a dome-shaped ceiling.—*Med. Press.*

